# Inhibiting Human and *Leishmania* Arginases Using *Cannabis sativa* as a Potential Therapy for Cutaneous Leishmaniasis: A Molecular Docking Study

**DOI:** 10.3390/tropicalmed7120400

**Published:** 2022-11-26

**Authors:** Aicha Assouab, Hajar El Filaly, Khadija Akarid

**Affiliations:** Biochemistry, Biotechnology and Immunophysiopathology Research Team, Health and Environment Laboratory, Ain Chock Faculty of Sciences, Hassan II University of Casablanca, Casablanca 20100, Morocco

**Keywords:** *Cannabis sativa*, *Leishmania* arginase, human arginase, cannabinoids, molecular docking

## Abstract

Cutaneous leishmaniasis (CL), a vector-borne parasitic disease caused by the *Leishmania* protozoan, is a serious public health problem in Morocco. The treatment of this disease is still based on pentavalent antimonials as the primary therapy, but these have associated side effects. Thus, the development of effective, risk-free alternative therapeutics based on natural compounds against leishmaniasis is urgent. Arginase, the key enzyme in the polyamine biosynthetic pathway, plays a critical role in *leishmaniasis* outcome and has emerged as a potential therapeutic target. The objective of this study was to test *Cannabis sativa*’s phytochemical components (cannabinoids and terpenoids) through molecular docking against *Leishmania* and human arginase enzymes. Our results showed that delta-9-tetrahydrocannabinol (THC) possessed the best binding energies of −6.02 and −6.35 kcal/mol with active sites of *Leishmania* and human arginases, respectively. Delta-9-THC interacted with *Leishmania* arginase through various amino acids including His139 and His 154 and linked to human arginase via His 126. In addition to delta-9-THC, caryophyllene oxide and cannabidiol (CBD) also showed a good inhibition of *Leishmania* and human arginases, respectively. Overall, the studied components were found to inhibit both arginases active sites via hydrogen bonds and hydrophobic interactions. These components may serve as therapeutic agents or in co-administrated therapy for leishmaniasis.

## 1. Introduction

Leishmaniasis is a parasitic infection caused by protozoan parasites that belong to the *Leishmania* genus and are transmitted to humans through an infected phlebotomine sandfly bite. This infection is associated with the following clinical forms: cutaneous leishmaniasis (CL), visceral leishmaniasis (VL), and mucocutaneous leishmaniasis (MCL) [1]. In fact, CL, the least severe clinical form of leishmaniasis, causes various types of skin lesions including ulcers, ulcero-crusted lesions, and nodular ones that are located in uncovered areas of the human body and leave disfiguring scars [2,3].

About 700,000 to 1 million new cases are reported each year worldwide [1]. CL is considered to be a public health problem especially in North African countries including Morocco and is induced by three species: *Leishmania major*, *L. tropica*, and *L. infantum* [4]. In the absence of a vaccine, the treatment of CL in Morocco is still based on pentavalent antimonials as the primary therapy. Furthermore, other molecules such as amphotericine B, pentamidine, paromomycin, and miltefosine were used as alternative therapies. Nevertheless, the use of these drugs is associated with many side effects such as relapses, high toxicity, and the emergence of drug-resistant parasitic strains [5]. Thus, and for better Leishmaniasis control, the design of an efficient anti-leishmanial drug must focus on the development of natural, less toxic, and cost-effective drugs with greater availability to low-income populations.

The parasite life cycle involves two stages: promastigote and amastigote. The promastigote-insect stage is characterized by an extracellular and flagellated form that is found in the vector’s gut. On the other hand, during the amastigote-vertebrate stage, the amastigote resides in mammalian cells, especially inside macrophages [6]. Interestingly, these immune cells are not only the replicative niche of *Leishmania*, but are also the anti-leishmanial effector cells [7]. To perform these contrasting functions, macrophages are characterized by functional plasticity and adopt different effector states. Through the influence of their microenvironment, naïve macrophages (M0) are activated and polarized toward “classically activated” (M1) or “alternatively activated” (M2) with different disease outcomes [8,9]. In these two types of macrophages, arginine is a key amino acid for two competing metabolic pathways: arginine can be converted to ornithine by arginase or alternatively to the anti-leishmanial agent nitric oxide (NO) by inducible nitric oxide synthase [10]. Classical M1 are activated upon Th1 cytokine exposure and contribute to the increased production of reactive oxygen species (ROS), including superoxide, hydrogen peroxide, hydroxyl radicals, and NO, which are crucial to the elimination of intracellular pathogens such as *Leishmania* due to their higher microbicidal capacity [9,11,12,13]. In contrast, Th2 cytokines induce the M2 profile, which is characterized by polyamine biosynthesis via activation of the arginase enzyme. This reaction is ensured through the bioconversion of L-arginine into L-ornithine and urea in the presence of manganese atoms. In fact, urea and L-ornithine are beneficial to intramacrophage *Leishmania* proliferation and disease progression [14,15,16,17].

An increased arginase activity associated with a decreased nitric oxide production is involved in leishmaniasis. Interestingly, *Leishmania* expresses arginase, and its activity has been shown to play an essential role in parasitic vital functions such as polyamine production and infectivity [18,19]. Arginase is also expressed in mammals; two of its isoforms were discovered: the hepatic arginase (ARG-1) and the extra-hepatic arginase (ARG-2), which is highly expressed in the kidney and prostate [20]. Host arginase (ARG-1) is induced by *Leishmania*, leading in turn to polyamine synthesis and thereby reinforcing *Leishmania*’s proliferation [16]. Consequently, both parasitic and host arginases are required for parasite growth and survival. Indeed, previous studies showed that patients with CL exhibited higher levels of Arg-1 in plasma and dermal lesions and demonstrated that there was a positive correlation of the CL severity with the host arginase level [9,21,22,23]. Using murine leishmaniasis models, studies have also demonstrated an increased arginase expression in susceptible BALB/c mice, which was associated with Th-2 cell response and a higher parasitic burden, resulting in the disease severity [17,24]. Moreover, the inhibition of host arginase was translated by a remarkable decrease in the parasitic load in infected mice [17,25,26]. Lee et al. also showed that a non-healing strain of *L. major* was efficiently phagocytized by M2 macrophages in vitro and in vivo. However, a strain that produced self-healing lesions was less phagocytosed by M2 macrophages. The authors stated that the preferential infection of M2 cells played a crucial role in the severity of the cutaneous disease [27]. Collectively, these studies suggested that both parasite and host arginases play a crucial role in the balance of life and death of *Leishmania* and underline the importance of arginase pathway as a potential and promising therapeutic target.

*Cannabis sativa* (*C. sativa*) is an annual plant belonging to the *Cannabaceae* family that is cultivated in different regions around the world and is used for many medicinal purposes [28,29]. *C. sativa* synthetizes several phytochemical compounds called cannabinoids, terpenoids, flavonoids, and alkaloids [30]. These natural components are the most identified in *C. sativa* and their production is impacted by specific environmental conditions such humidity, temperature, and others [29]. The phytocannabinoids found in organic extracts of Moroccan *C. sativa* are delta-9-tetrahydrocannabinol (THC) and cannabidiol (CBD), both of which belong to the most studied cannabinoids [31,32]. In addition to the phytocannabinoids, terpenoids such as α-humulene, β-caryophyllene, β-myrcene, caryophyllene oxide, α-pinene, decane, and limonene were described in different *C. sativa* essential oils [33,34]. In vitro, *C. sativa* extracts and active components showed an antimicrobial potential against pathogenic bacteria, fungus, and parasites, including *Leishmania* [35,36]. In addition, in vivo studies on a CL hamster model showed that this plant extract exhibited remarkable anti-leishmanial and healing potentials. Interestingly, the application of a *C. sativa*-based cream on CL-associated skin lesions promoted the healing process [37]. Nonetheless, these overall studies did not go further to determine *C. sativa*’s possible targets and mechanisms of action.

In light of these results and due to the existing interplay between host and parasite arginine, as well as the importance of polyamine metabolism in the outcome of *Leishmania* infections, our aim was to highlight the molecular interactions of cannabinoids and terpenoids with both arginases through molecular docking. Although this was an in silico work, it will contribute to the discovery of new natural and effective compounds against CL.

## 2. Materials and Methods

### 2.1. Collection and Preparation of C. sativa Ligands

The *C. sativa* main components used in this study were: delta-9-tetrahydrocannabinol (delta-9-THC), cannabidiol (CBD), caryophyllene oxide, beta-caryophyllene, α-pinene, α-humulene, myrcene, and limonene. Glucantime^®^ was used as our reference drug because it is used as the first-line cutaneous leishmaniasis treatment in Morocco. These ligands were obtained from published research papers and downloaded from the PubChem database (Table 1) in an SDF file and converted to a 3D PBD file using Open Babel GUI (version 2.4.1).

### 2.2. Retrieval of Arginases’ 3D Structures

The crystal structure of *Leishmania mexicana* arginase (*Lm*Arg) (accession ID: 4ITY) and human arginase (h-Arg) (accession ID: 3kv2) were obtained from the RCSB Protein Data Bank in PDB format (https://www.rcsb.org, accessed on 13 August 2022) [38,39]. It was demonstrated that *Leishmania* species’ arginases exhibited highly conserved DNA and amino acid sequences [40]. For further corroboration, we blasted the protein sequences of *L*. *mexicana* and the CL-induced species in Morocco (*L. major* and *L. tropica*), which showed 95% similarities. In addition, the 5% variation did not impact the active site’s amino acids.

### 2.3. Molecular Docking

A molecular docking study was performed using Auto Dock tools (ADT) (version 1.5.7) to analyze the conformations of the major phytochemical compounds.

Then, the proteins were prepared independently for docking by deleting all non-standard residues and water molecules and adding polar hydrogen atoms and Kollman charges to the macromolecule. The grid box dimensions were set to x = 50, y = 50, z = 40 and x = 70, y = 70, z = 60 with a grid spacing of 0.375 Å. A grid center was regulated at x = 15.612, y = −13.729, z = −5.583 and x = 21.314, y = −14.067, z = −3.497 around the active sites of the *Leishmania* and human arginase, respectively.

For each compound, 100 docking runs were performed using the Lamarckian genetic algorithm (LGA) to search for the best binding pose. Then, the optimal conformations were analyzed using Biovia Discovery Studio 2021 software.

## 3. Results

In this study, we performed a molecular docking of the main *C. sativa* compounds with *Leishmania* and human arginase proteins in order to visualize the mechanism by which the binding was carried out. The referent anti-leishmanial drug Glucantime^®^ was used as a control.

### 3.1. C. sativa Components and Leishmania Arginase: Molecular-Interaction Analysis

**Figure 1:**
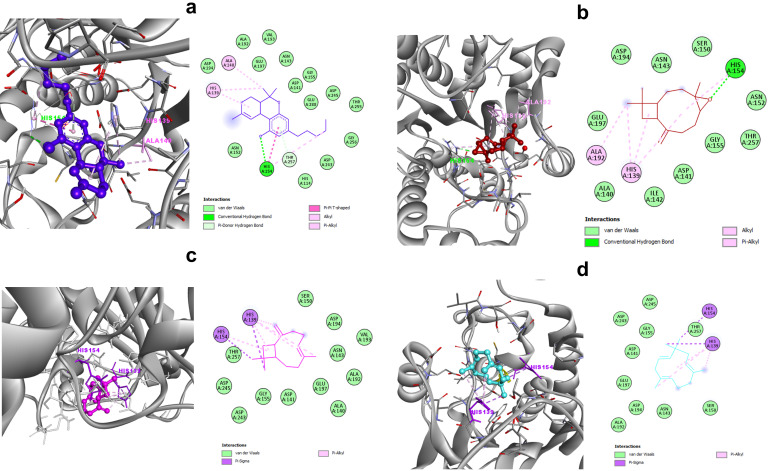
Molecular docking of ligands with *Leishmania* arginase. Interaction of delta-9-THC (**a**), caryophyllene oxide (**b**), beta-caryophyllene (**c**), and α- humulene (**d**) with the *Lm*Arg protein and its linked amino acid residues.

The docking results are presented in Table 2. Among the tested ligands, while interacting with *Lm*Arg, the delta-9-THC, caryophyllene oxide, beta-caryophyllene, and α-humulene showed the best binding scores of −6.02, −5.88, −5.79, and −5.55 kcal/mol, respectively. Although limonene had a score of −4.49 kcal/mol, the lowest ligand–protein affinity was in fact assigned to Glucantime^®^ at −4.30 kcal/mol. These results were correlated with inhibition constants ranging between 38.63 µM for delta-9-THC and 702.95 µM for Glucantime^®^. Delta-9-THC has established conventional hydrogen bonds with HIS 154 and hydrophobic interactions with HIS 139 and ALA 140. The pi–donor hydrogen-bond interaction was also observed for TRH 257. There were also 14 amino acids (HIS 114, ASP 141, ASN 143, ASN 152, GLY 155, ALA 192, VAL 193, ASP 194, GLU 197, ASP 243, ASP 245, THR 255, GLU 256, and GLU 288) involved in weak van der Walls interactions (Figure 1a). On the other hand, caryophyllene oxide fit in the active site of *Lm*Arg and interacted with HIS 154 via conventional hydrogen bonds and with two other amino acids (HIS 139 and ALA 192) via hydrophobic interactions. Moreover, ALA 140, ASP 141, ILE 142, ASN 143, SER 150, ASN 152, GLY 155, ASP 194, GLU 197, and THR 257 were associated with target protein via the van der Waals forces (Figure 1b). Beta-caryophyllene formed pi–sigma interactions with HIS 139 and HIS 154, as well as van der Waals interactions with other residues (ALA 140, ASP 141, ASN 143, SER 150, GLY 155, ALA 192, VAL 193, ASP 194, GLU 197, ASP 243, ASP 245, and THR 257), as shown in the 2D interactions plot (Figure 1c). Similarly, on the active site of parasite arginase, α-humulene showed hydrophobic interactions with the same amino acids as beta-caryophyllene. Furthermore, van der Waals forces were observed for ASP 141, ASN 143, SER 150, GLY 155, ALA 192, ASP 194, GLU 197, ASP 243, ASP 245, and THR 257 (Figure 1d).

### 3.2. C. sativa Components and Human Arginase: Molecular-Interaction Analysis

**Figure 2:**
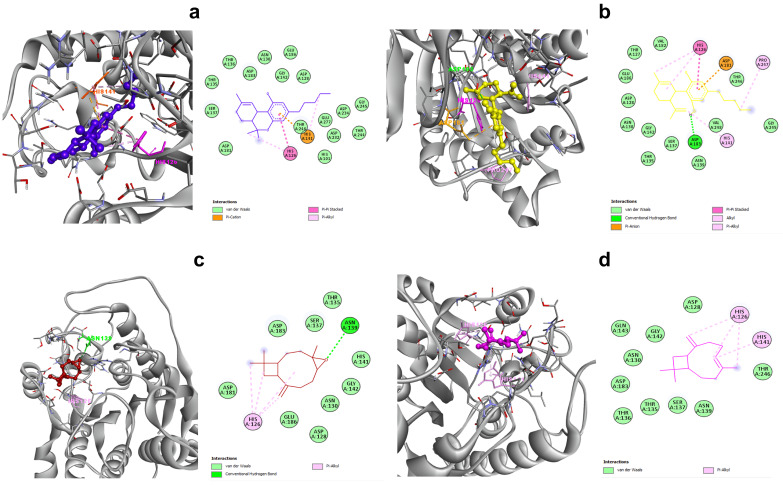
Visualization of interactions between various *C. sativa* compounds and human arginase. Interaction of delta-9-THC (**a**), CBD (**b**), caryophyllene oxide (**c**), and beta-caryophyllene (**d**) with the h-Arg protein and its linked amino acid residues.

The docking results also indicated that among the tested ligands, delta-9-THC fit in the active site of human arginase with a binding energy of −6.35 kcal/mol and an inhibition constant (Ki) of 21.97 µM through a pi–cation interaction with HIS 141, a pi–pi stacked interaction with HIS 126, and van der Waals forces with 16 other amino acids (Figure 2a). The docked structure of cannabidiol (CBD) revealed a negative binding energy of −5.60 kcal/mol and an inhibition constant of 78.86 µM. LmArg and CBD interactions resulted in conventional hydrogen bond, pi–anion, pi–pi stacked, and pi–alkyl interactions with ASP 183, ASP 181, HIS 126, HIS 141, and PRO 247, respectively. Furthermore, van der Waals forces were also involved in associations with THR 127, ASP 128, ASN 130, THR 135, SER 137, ASN 139, GLY 142, VAL 182, GLU 186, GLY 245, THR 246, and VAL 248 (Figure 2b). The predicted binding energy and inhibition constant of caryophyllene oxide were −5.37 kcal/mol and 115.51 µM, respectively. This ligand showed interactions with ASN 139 via conventional hydrogen bonds and with HIS 126 through a hydrophobic interaction. Furthermore, 9 amino acids (ASP 128, ASN 130, THR 135, SER 137, HIS 141, GLY 142, ASP 181, ASP 18, and GLU 186) participated via van der Waals forces (Figure 2c). Beta-caryophyllene revealed a binding energy of −5.47 kcal/mol and an inhibition constant of 97.47 µM by interacting with the active site of the target enzyme. Beta-caryophyllene’s interaction with h-Arg residues showed that HIS 126 as well as HIS 141 formed alkyl hydrophobic links, while van der Waals forces (ASP 128, ASN 130, THR 135, THR 136, SER 137, ASN 139, GLY 142, GLN143, ASP 183, THR 246) formed links with phytochemical compounds (Figure 2d).

Moreover, among the selected phytochemical compounds of *C. sativa*, delta-9-tetrahydrocannabinol showed the best binding energy with both enzymes, followed by caryophyllene oxide for *Lm*Arg and cannabidiol for h-Arg.

## 4. Discussion

Cutaneous leishmaniasis is a neglected tropical infection that requires proper treatment and close monitoring [40]. The drugs used for leishmaniasis treatment are still based on pentavalent antimonials (Glucantime^®^ and Pentostam^®^), which have severe side effects [5]. Hence, natural compounds derived from plants are characterized by their efficiency, low toxicity, and cost, and are associated with minor side effects. In fact, these natural molecules have been exploited as alternative anti-leishmanial agents [5]. Previously, it was reported that a crude methanolic extract of *C. sativa* with a high amount of phenolic compounds possessed an in vitro growth-inhibition effect on the *L. major* parasite [41]. Furthermore, three *C. sativa* essential oils composed mainly of myrcene, α-pinene, and e-caryophyllene induced an in vivo protective effect in *L. tropica*-infected mice [42].

CL is characterized by localized skin lesions in which arginase is upregulated and involved in *Leishmania* proliferation [21,43]. *Leishmania* arginase represents the first enzyme involved in polyamine biosynthesis; its lack blocked *L*. *mexicana* as well as *L. major* growth, but it was restored by adding polyamines [18,44]. Consequently, various studies proved that flavanols such as quercetin as well as verbascoside were effective against extracellular promastigote and intracellular amastigote forms of *L. amazonensis* by inhibiting parasitic arginase [45,46,47]. Similarly, 2(S)-amino-6-boronohaxanoic acid (ABH) blocked *Leishmania* and/or human arginase activities [38,43]. Interestingly, our in silico study, which used both parasitic and human arginases, highlighted for the first time the molecular interactions between phytochemical components of *C. sativa* and the active site of these enzymes. Therefore, our docking interaction results for the *L. mexicana* arginase active site indicated that delta-9-THC and three other selected ligands interacted with the amino acids HIS 139, HIS 154, ALA 192, and ALA 140. HIS 139 belongs to the amino acids involved in bridging with Mn^2+^ [48,49]. This latter interaction with nor-NOHA blocked arginase activity, as demonstrated previously [50]. Therefore, this metal bridging is necessary for arginase activity, which led us to suggest that interactions between delta-9-THC and HIS 139 might be responsible for the inhibition of this target enzyme. Furthermore, HIS 154 is directly involved in the L-arginine substrate and binds to *L. mexicana* as well as *L. amazonensis* arginases [48,51]. Consequently, interaction between HIS 154 and delta-9-THC might be responsible for arginase inhibition. Another compound, phenylacetamide, as well as caryophyllene oxide, interacted with the ALA 192 amino acid, which resulted in the inhibition of *Leishmania* arginase activity [49]. In addition, *Leishmania* arginases sequences, mainly in the active site, possessed a high degree of conservation [47]. Thus, this result could be exploited in the context of different *Leishmania* species such as *L. major*, *L. tropica*, and *L. infantum*, which are responsible for CL in Morocco.

The human arginase amino acid residues involved in our protein ligand links were HIS 126, ASN 139, HIS 141 ASP 181, and PRO 247; some of these amino acids are involved in the active site of human arginase [52,53]. Among the studied cannabinoids, delta-9-THC showed good inhibitory activity, as did CBD. In addition, they shared some properties such as strong antioxidant, anti-inflammatory, antimicrobial, and immunomodulatory activities [54,55,56]. Our molecular analysis demonstrated that caryophyllene oxide was linked to human arginase by different residues that included ASN 139 and HIS 126. Previously, cinnamide derivatives were also reported to inhibit mammalian arginase through interactions with HIS 126, HIS 141, and other target pocket residues [57]. Moreover, lignanamides, which are new compounds that were isolated from *C. sativa*, were reported as natural inhibitors of bovine arginase [58]. In addition, delta-9-THC and beta-caryophyllene involved different residues while interacting with LmArg and h-Arg. Interestingly, the cannabinoids and terpenoids selected for this study were found to inhibit both human and *Leishmania* arginases’ active sites via hydrogen bonds as well as via hydrophobic interactions. The interplay of the host, parasite arginine, and polyamine metabolism is decisive in the outcome of *Leishmania* infections. In addition, the *Leishmania* parasite has the ability to modulate the host arginase activity and therefore the immune response [10]. Therefore, studies suggested that inhibition of the parasite arginase alone may not be a sufficient therapeutic strategy. Indeed, although the infectivity level was diminished, the arginase-deficient mutants in different *Leishmania* species were still able to establish infections [10,44,59].

## 5. Conclusions

Since CL is still a public health problem in low-income and developing countries, the discovery of an efficient, less toxic, and accessible therapy is a necessity. The present in silico study was the first to investigate *C. sativa*’s selected constituents as selective inhibitory agents for parasitic as well as host arginases, which play an important role in this parasitic infection pathology. Interestingly, THC showed a great inhibitory potential for both species’ enzymes and will allow a better control of leishmaniasis.

Although these docking results were interesting and promising, they require in vitro as well as in vivo experiments to corroborate and develop new approaches to leishmaniasis treatment and control.

## Figures and Tables

**Table 1:** Selected phytochemical compounds characteristics of *C. sativa*.

Ligands	Characteristics	Structures
Delta-9-Tetrahydrocannabinol	MW:314.5 g/mol MF: C21H30O2 PubChem ID: CID 16078	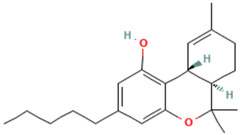
Cannabidiol (CBD)	MW:314.5 g/mol MF: C21H30O2 PubChem ID: CID 644019	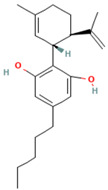
Caryophyllene Oxide	MW:220.35 g/mol MF: C_15_H_24_O PubChem ID: CID 1742210	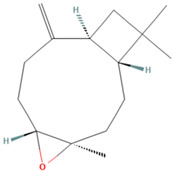
Beta-Caryophyllene	MW:204.35 g/mol MF: C_15_H_24_ PubChem ID: CID 5281515	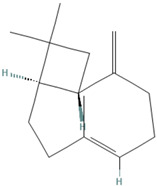
α-Pinene	MW:136.23 g/mol MF: C_10_H_16_ PubChem ID: CID 6654	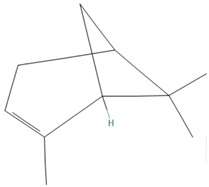
α-humulene	MW:204.35 g/mol MF: C_15_H_24_ PubChem ID: CID 5281520	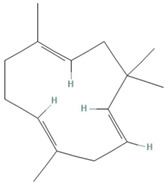
Myrcene	MW:136.23 g/mol MF: C_10_H_16_ PubChem ID: CID 31253	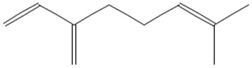
Limonene	MW: 136.23 g/mol MF: C_10_H_16_ PubChem ID: CID 22311	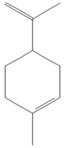
Glucantime^®^ (meglumine antimoniate)	MW:365.98 g/mol MF: C_7_H_18_NO_8_Sb PubChem ID: CID 64953	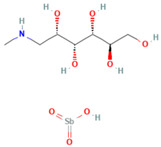

MW, molecular weight; MF, molecular formula.

**Table 2:** Predicted values of binding energies and inhibition constants linking the ligands and the target proteins.

Ligand	Arginase			
	*Leishmania* (4ITY)		Human (3kv2)	
	Binding Energy (kcal/mol)	Ki (µM)	Binding Energy (kcal/mol)	Ki (µM)
Delta-9-Tetrahydrocannabinol	−6.02	38.63	−6.35	21.97
Cannabidiol (CBD)	−5.41	108.33	−5.60	78.86
Caryophyllene Oxide	−5.88	49.07	−5.37	115.51
Beta-Caryophyllene	−5.79	56.56	−5.47	97.47
α-Pinene	−4.58	441.46	−4.90	254.71
α-humulene	−5.55	85.30	−5.25	140.93
Myrcene	−4.79	307.93	−4.88	266.94
Limonene	−4.49	507.25	−4.61	416.05
Glucantime^®^	−4.30	702.95		

Ki: inhibition constant.

## Data Availability

The data presented in this study are available on request.
